# Community Perspective of Mental Health and Mental Health Care Among Rural Population in Faridabad, Haryana: A Qualitative Study

**DOI:** 10.7759/cureus.82332

**Published:** 2025-04-15

**Authors:** Aninda Debnath, Harshal Ramesh Salve

**Affiliations:** 1 Department of Community Medicine, Maulana Azad Medical College, New Delhi, IND; 2 Department of Epidemiology and Public Health, Centre for Community Medicine, All India Institute of Medical Sciences, New Delhi, IND

**Keywords:** community perception, focus group, grounded theory, india, mental health, qualitative research, stigma, traditional healers

## Abstract

Background

Mental illness constitutes a significant and growing public health concern in India, with limited access to care and pervasive stigma contributing to low treatment rates. There is limited qualitative research examining community-level perspectives on mental health in rural Indian settings. The objective of the study was to study community perspectives on mental illness and mental health services in the district of Faridabad.

Methods

A qualitative study was conducted using five focus group discussions (FGDs) involving community members and Accredited Social Health Activists (ASHAs) across four primary health centers (PHCs) in Faridabad. Data were collected through semi-structured discussions, transcribed, and analyzed using grounded theory with open, axial, and theoretical coding. Rigor was ensured through triangulation, field notes, and iterative analysis until data saturation was achieved.

Results

Grounded theory analysis identified six themes contributing to community perceptions and help-seeking for mental illness: (1) mental illness perceived as abnormal behavior; (2) strong stigma and fear of social contagion; (3) attribution to psychosocial stressors such as tension and family conflict; (4) preference for traditional healers over medical professionals; (5) structural barriers to accessing formal mental health care; and (6) stigma-related avoidance of professional help. These categories formed a cyclical explanatory model in which cultural beliefs, structural limitations, and social stigma perpetuate reliance on informal care and delay engagement with formal mental health services.

Conclusion

Community perceptions in Faridabad reflect a complex interplay of cultural, social, and structural factors that delay engagement with formal mental health care. Interventions must address stigma, improve service accessibility, and incorporate culturally sensitive public education to improve mental health outcomes in similar Indian contexts.

## Introduction

Mental health is a crucial component of overall health, yet mental illness remains a significant challenge globally. In 2017, mental disorders were the second leading cause of disease burden in terms of years lived with disability (YLDs) and the sixth leading cause of disability-adjusted life-years (DALYs) worldwide [1]. In India, 197.3 million people were estimated to be living with mental illness, including 45.7 million with depressive disorders and 44.9 million with anxiety disorders. The contribution of mental illness to total DALYs in India increased from 2.5% in 1990 to 4.7% in 2017, reflecting the growing burden [2].

Despite the launch of the National Mental Health Programme (NMHP) in 1982, India faces a substantial treatment gap, ranging from 28% to 83% across various mental health conditions [3,4]. Recent studies, such as one by Sagar et al., highlight a gap as high as 95%, with barriers including low perceived need, attitudinal factors, and stigma [5]. In traditional societies of India, mental illness is often conceptualized through supernatural beliefs or alternate systems of medicine rather than biomedical explanations [6].

Stigma is another major roadblock to treatment-seeking and contributes to the huge burden of mental illness [7]. Thornicroft et al. identified three elements of stigma: ignorance, prejudice or discrimination, and misinformation [8]. NMHS shows that 80% of the persons suffering from mental illness had not received any treatment despite the presence of illness for more than 12 months [9].

Despite the growing recognition of mental health as a critical public health issue, there remains a paucity of qualitative research exploring the community’s perspective on mental health and mental health care in specific Indian regions. Quantitative approach often misses the rich, contextual nuances of how individuals understand and interact with mental health services. Understanding community attitudes and beliefs is crucial for developing targeted interventions that address the specific barriers to mental health care utilization. The present study aims to fill this gap by exploring community perspectives on mental health and mental health care in Faridabad district, Haryana, India. The objective of this study was to examine community perspectives on mental illness and mental health services in the district of Faridabad, Haryana, through a qualitative study.

## Materials and methods

Setting

The study was conducted in villages covered by four primary health centers (PHCs) selected based on the availability of mental health services. Two PHCs had the provision of weekly psychiatric services by psychiatrists through Outreach Specialty OPD (ORSO) clinics. The other two PHCs had no specialized mental health clinics, and mental health care services were solely provided by the Medical Officer-in-Charge (MOIC). This comparative approach allowed us to capture community perspectives from areas with different levels of mental health service availability.

Community engagement and recruitment

Community engagement was facilitated by health workers from the respective PHCs. Initial visits were made to introduce the study to the MOICs and health workers. Recruitment was conducted during planned village visits, where residents were approached individually and invited to participate in focus group discussions (FGDs). We employed purposive sampling to ensure the inclusion of diverse perspectives relevant to the study objectives. Information and consent forms were provided in Hindi, ensuring accessibility to all participants. The study was conducted over a two-month period, from May to June 2019.

Data collection

Data were collected through five FGDs. FGDs were designed to explore community perceptions of mental illness and awareness of available mental health services (governmental, private, and traditional healers). Two FGDs were conducted with Accredited Social Health Activists (ASHAs), while three FGDs were held with adult community volunteers. Separate FGDs were organized for men and women in recognition of local cultural sensitivities. Topics discussed included community responses to mental illness, local concepts of mental illness, and the role of key service providers in mental health care. All FGDs were audio recorded with participants' consent, and the recordings were made by the author (AD).

Data management and analysis

Data were analyzed using the principles of constructivist grounded theory through an iterative process of open, axial, and theoretical coding. Initially, open coding was done, where transcripts were reviewed line by line to identify key concepts and actions embedded in participants' narratives. In the subsequent stage of axial coding, related codes were grouped into broader conceptual categories, allowing for the identification of core patterns and relationships across participant groups. These categories were then further refined through theoretical coding, in which connections between the categories were explored to construct an explanatory model that reflected how community members understand and respond to mental illness. Data collection and analysis occurred simultaneously, allowing emerging insights to inform subsequent discussions and coding decisions. The constant comparative method was employed throughout, comparing data within and across focus groups to refine categories and ensure analytical depth. Analysis continued until theoretical saturation was achieved.

Quality control

Several techniques were employed to ensure rigor. Field notes were maintained separately and regularly reviewed by the research team to ensure the accuracy of the transcriptions and the integrity of the data. Data saturation was reached, as no new information emerged during the final FGDs. Triangulation was achieved through the inclusion of different data sources (FGDs with both ASHAs and community members), enhancing the validity of the findings. Unusual or unexpected responses were further explored to ensure comprehensive coverage of the community's perspectives.

Ethical considerations

Ethical clearance was obtained from the Institute Ethics Committee of All India Institute of Medical Sciences (AIIMS), New Delhi. All participants were provided with information sheets in Hindi detailing the study's objectives, procedures, expected benefits, and potential risks. Participants were informed that their involvement was voluntary and that they could withdraw at any time without any penalty. Written informed consent was obtained from all participants prior to their involvement in the study.

## Results

The study’s findings, derived from five FGDs, two with ASHA workers and three with adult community members, provide a comprehensive understanding of how mental illness is perceived within the community and the challenges faced in seeking mental health care. Each FGD included seven to nine participants, with an average duration of 52 minutes. The details of the five FGDs, including participant characteristics and discussion durations, are presented in Table 1. The analysis generated a grounded theory illustrating how culturally mediated understandings of mental illness, combined with systemic and social barriers, contribute to a recurring cycle of delayed formal care and reliance on traditional healing practices.

**Table 1:** Participant profile and duration of focus group discussions ASHA = Accredited Social Health Activist, FGD = focus group discussion, SD = standard deviation

Details of the FGD	Total participants	Study participants	Mean age in years ± SD	Duration of FGD (minutes)
FGD1	7	Community members (male)	38 ± 6	47
FGD2	9	Community members (female)	39 ± 6.2	40
FGD3	8	Community members (male)	43 ± 5.5	52
FGD4	9	ASHA workers	42 ± 5.8	64
FGD5	8	ASHA workers	40 ± 6.4	57

Perceptions of mental illness as abnormal behavior

Mental illness is primarily understood through observable behavioral deviations in the community. The participants described mental illness as a disturbance in the brain, which manifests through actions that are seen as inappropriate or deviant. Common descriptions included self-talk, wandering aimlessly, inappropriate clothing, and even aggressive tendencies, such as sudden outbursts or attempts to physically harm others. The community’s perception of mental health is behavior-centric, focusing on external actions rather than underlying causes or conditions.

Participants emphasized that mental illness is synonymous with erratic behavior. For example, the terms "Pagal" (mad) and "Baad-dimag" (shaken brain) were commonly used to describe individuals with mental health problems, reinforcing the notion that mental illness is something to be feared and avoided.

"Brain gets shaken, that is mental illness. We get different thoughts in our mind. Sometimes, we think of something and sometimes something else." (Participant 2, FGD 5)

"They behave like madmen. He keeps wearing half clothes and takes off his own clothes." (Participant 4, FGD 1)

This behavior-centric framing of mental illness significantly shapes how mental health conditions are recognized and treated. The focus on visible symptoms limits the community’s understanding of mental illness to extreme cases, often overlooking conditions like depression, anxiety, or other less overt forms of psychological distress. As a result, those who do not exhibit these behaviors may not be perceived as mentally ill, delaying both recognition and treatment of their condition.

Stigma and fear of social contagion

The stigma surrounding mental illness in the community is profound, resulting in the social isolation of individuals suffering from mental health conditions. Mental illness is not only viewed as abnormal but also as something potentially dangerous or contagious, with participants expressing fears that associating with someone who is mentally ill could result in harm or "catching" the illness. This fear leads to individuals with mental illness being ostracized, both by their families and by the larger community.

A strong theme in the data was the belief that mental illness brings shame and dishonor to families, particularly in relation to marriage prospects. Families with mentally ill members are often considered unfit for marriage alliances, further isolating the individuals affected by mental illness. The social implications of mental illness extend beyond the individual, affecting entire households and reinforcing the stigma associated with seeking help.

"Our parents say that we have to keep distance from the lunatics. They even say that such people can kill." (Participant 2, FGD 2)

"If there is a mentally ill patient in some house, who would like to get married in that house? No one will." (Participant 4, FGD 1)

The social stigma manifests not only in avoidance and isolation but also in mockery and disrespect. Individuals with mental illness are laughed at and treated as objects of ridicule. This dehumanization of mentally ill individuals perpetuates the cycle of isolation and discourages families from acknowledging or seeking treatment for their loved ones.

"Many people laugh at them. Some think that by touching, the disease can spread, so they stay away." (Participant 3, FGD 1)

Stigma also discourages help-seeking behaviors, as families fear social ostracism if their relative's mental health condition becomes public knowledge. As a result, many choose to hide the illness, further delaying or avoiding professional care.

Psychosocial attribution of mental illness

The community’s understanding of the causes of mental illness is predominantly psychosocial, with stress and emotional trauma identified as the primary contributors. Participants frequently associated mental illness with tension, family conflicts, or emotional distress stemming from personal life events such as romantic relationship issues, household quarrels, or loss. The belief that life events trigger mental health problems was prevalent, and many participants provided examples of individuals who had become mentally ill following significant stress or trauma.

There is a strong association between mental illness and relationship breakdowns, with participants recounting stories of individuals whose mental health deteriorated after being abandoned by a partner or spouse. These emotional events are seen as leading to social withdrawal, sadness, and eventual mental illness.

"The biggest cause of mental illness is tension. Tension of something causes mental illness." (Participant 2, FGD 4)

"If there is a quarrel going on in someone's house, gradually, the man starts getting unhappy. He gets mental illness." (Participant 3, FGD 5)

Participants also described mental illness as the result of overthinking or worrying excessively about unresolved issues. This cognitive explanation for mental illness is consistent with traditional beliefs that emotional and psychological stressors can affect a person’s mental balance.

"His girlfriend left him. After that, he started living silently. He did not talk to the people." (Participant 1, FGD 3)

While psychosocial stress is acknowledged as a major cause of mental illness, there is little understanding of the biological or genetic factors that may contribute to mental health problems. This limited understanding of mental illness as purely a reaction to stress further reinforces the community’s reliance on traditional healers, as they believe mental health issues can be resolved through non-medical interventions.

Reliance on traditional healers as a first line of treatment

Traditional healers play a central role in the community’s mental health care-seeking behavior. When individuals or families recognize signs of mental illness, their first step is often to consult a traditional healer rather than a formal health care provider. Participants cited multiple reasons for this preference, including convenience, cultural beliefs, and the perception that they will provide faster results than doctors. The community believes that traditional healers possess spiritual or mystical knowledge that can address mental health issues, particularly those they perceive as being caused by external or supernatural forces.

"Treatment is fast when anyone seeks care from *Baba* (traditional healer). It gets too late to get treatment from the doctor." (Participant 6, FGD 1)

"First, we showed it to *Baba*, but there was no benefit. Then we took him to the medical, but medical is very far away, and there is a very long line. So again, he was taken to *Baba*." (Participant 7, FGD 3)

While many participants acknowledged that formal medical treatment might eventually be necessary, they often return to traditional healers for further treatment if medical care does not provide immediate relief. The inconsistencies in medical care, such as long waits, form-filling, and complex procedures, make traditional healers an appealing alternative despite their lack of scientific validation.

Structural barriers to formal mental health care

The community’s frustration with formal health care services was a recurring theme, with participants describing several barriers to accessing mental health services. Long distances, extensive waiting periods, medication shortages, and bureaucratic procedures were among the most frequently cited challenges. Many participants noted that government hospitals are often overcrowded, with long lines and insufficient resources, leading them to turn to traditional healers instead.

"Medical is very far away, and there is a very long line." (Participant 7, FGD 3)

"People do not want to go to government hospital. The medicines are never available." (Participant 3, FGD 1)

Even when participants sought formal medical treatment, they often encountered issues with continuity of care or lack of awareness about where to access specialized mental health services. This lack of a robust mental health infrastructure, combined with the cost of seeking care from private hospitals, discourages people from seeking professional help. As a result, they often return to traditional healers who are more familiar and accessible despite the limitations of such treatments.

Stigma in mental health help-seeking

The stigma attached to mental illness not only affects those diagnosed but also deters others from seeking treatment. Families and individuals fear the social consequences of being labeled as mentally ill, which include the risk of being ostracized or ridiculed by the community. This fear of social judgment prevents many from acknowledging mental health problems and delays help-seeking behavior. Even when families understand the need for professional care, the stigma surrounding mental illness often overrides their willingness to seek help from mental health professionals.

"If someone tries to talk with them, they behave rudely... paying attention will not be beneficial." (Participant 7, FGD 5)

"People also go to *Baba.* If *Baba* is doing right, why won't they go?" (Participant 1, FGD 4)

This theme underscores the dual challenge of addressing both the social stigma surrounding mental illness and the systemic barriers to accessing mental health care in the community. Without reducing the stigma attached to mental illness, interventions designed to improve mental health outcomes may be less effective, as people will continue to avoid formal health care out of fear of judgment.

**Figure 1:**
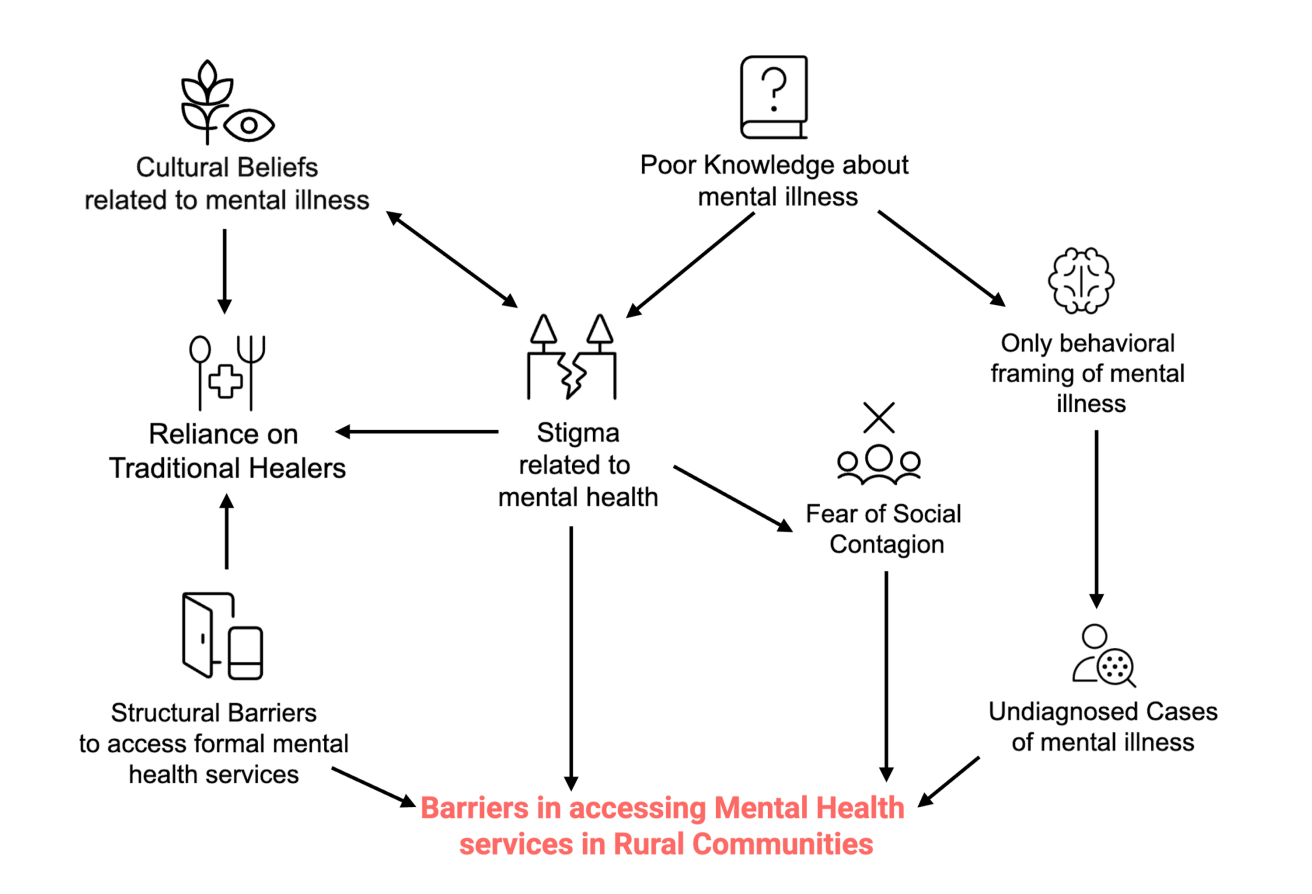
Conceptual model of stigma, structural barriers, and cultural beliefs influencing mental health help-seeking

These findings illustrate a self-reinforcing cycle in which community perceptions of mental illness, primarily defined by visible behavioral disturbances, combine with deep-rooted stigma, psychosocial attributions, and limited access to formal care to drive reliance on traditional healing practices. This cycle is further sustained by insufficient awareness of biomedical explanations, structural barriers such as distance and resource limitations, low confidence in navigating formal health systems, and persistent fears of social judgment. Together, these interconnected factors delay engagement with professional mental health services and perpetuate informal, culturally embedded responses to mental illness (Figure 1).

## Discussion

This study explored community perceptions of mental illness and mental health care in Faridabad, revealing a grounded theory that explains how sociocultural beliefs, stigma, and systemic barriers interact to delay formal treatment-seeking and reinforce reliance on traditional healing. Six key themes emerged: the behavioral framing of mental illness, fear of social contagion, attribution to psychosocial stressors, reliance on traditional healers, structural barriers to formal care, and stigma impeding help-seeking. These findings align with existing literature while offering new insights into the cyclical and self-reinforcing nature of mental health care avoidance in rural Indian settings.

In line with the study’s findings, mental illness in rural Indian communities is frequently perceived through behavior-centric lenses, where deviant or erratic behavior, such as self-talk or aggressive outbursts, is considered the primary indicator. This perspective overlooks less visible conditions like depression or anxiety, which do not manifest in overt behavioral changes, delaying diagnosis and treatment. Similar findings were reported by Jadhav et al. and Ganesh, who observed that this narrow framing of mental illness is widespread in India, particularly in rural areas [10,11].

The study highlights the deep stigma attached to mental illness, particularly the fear of social contagion, where not only the individual but their entire family risks social ostracism. This is especially relevant in cultural contexts like marriage, where the presence of mental illness can severely impact familial prospects. Mukherjee et al. and Raguram et al. similarly found that this fear of stigma leads to the concealment of mental illness and discourages families from seeking help [12,13]. The pervasive belief that mental illness "contaminates" social standing significantly reduces help-seeking behavior and limits community support, creating a cycle of secrecy and marginalization.

In this study, the community predominantly attributed mental illness to psychosocial stressors such as family conflicts, work pressure, or relationship breakdowns. Biological or genetic explanations were largely overlooked, a pattern also seen in studies by Benti et al. and Salve et al., where mental illness was perceived as a response to emotional stress rather than a medical condition [14,15]. This psychosocial framing often leads communities to rely on coping mechanisms or social interventions rather than medical treatment, reinforcing the belief that mental illness can be managed outside of the health care system. This points to the need for public health campaigns to educate communities on the multifactorial nature of mental illness, including biological factors.

The reliance on traditional healers as the first point of contact for mental health issues remains a significant barrier to accessing formal psychiatric care. This reflects cultural beliefs linking mental illness to supernatural causes, such as evil spirits or divine retribution, as noted in other studies [15-17]. Scarce mental health facilities in rural areas, combined with dissatisfaction with formal health care due to long distances, medication shortages, and inefficiencies, drive communities toward traditional healers, who are more accessible and culturally aligned [18]. Jadhav et al. and Mukherjee et al. report that these barriers delay or prevent treatment, reflecting broader distrust in public health care [10,12]. This highlights the need to integrate traditional healers into mental health frameworks and improve health care infrastructure by reducing barriers and ensuring consistent medication availability.

Stigma remains a central obstacle to help-seeking behavior, as families fear the social consequences of being labeled as mentally ill. Raguram et al. and Bӧge et al. [13,19] found that fear of social judgment and ostracism often prevent individuals from seeking psychiatric care, even when they recognize its necessity. This stigma is not limited to the individual but extends to the family, reinforcing a culture of secrecy and avoidance. Addressing this stigma will require both broad-based community education and targeted interventions aimed at reducing the fear of judgment and encouraging open discussion of mental health issues.

This study's use of FGDs allowed for rich qualitative insights into community perceptions, going beyond surface-level attitudes. The inclusion of both community members and ASHA workers ensured a comprehensive understanding of mental health issues from multiple perspectives. Additionally, the comparative approach between areas with varying levels of mental health service availability provides a nuanced view of how these services impact community attitudes. However, the study is limited by its focus on a single district, which may not be representative of other regions with different cultural or socioeconomic contexts. Additionally, cultural norms may have led participants to underreport certain beliefs or behaviors, especially regarding sensitive topics like mental illness. Finally, while the qualitative data offers deep insights, the study would benefit from complementary quantitative data to provide a broader perspective on the extent of these attitudes within the population.

Future research should explore how these findings apply to other regions of India, particularly those with different cultural or religious contexts. Expanding the geographical scope would provide a more comprehensive understanding of how community perceptions of mental illness vary across diverse settings. Engaging traditional healers in formal research could also provide deeper insights into their influence on mental health outcomes and how they can be integrated into formal health care systems.

## Conclusions

This study reveals the complex sociocultural and structural factors shaping community perceptions of mental illness in Faridabad, revealing six key interlinked categories: perception of abnormal behavior as mental illness, stigma and social contagion, psychosocial attribution, reliance on traditional healers, structural barriers, and stigma-related help avoidance. The reliance on traditional healers, fear of social contagion, and structural barriers to formal mental health care create a cycle of delayed help-seeking behavior. Culturally sensitive public health interventions, improved mental health infrastructure, and efforts to reduce the stigma associated with mental illness are essential for addressing these challenges.
